# Drying Kinetics of the Leaves From Three Varieties of Cassava (*Manihot esculenta* Crantz)

**DOI:** 10.1002/fsn3.71494

**Published:** 2026-01-28

**Authors:** Elivaldo Nunes Modesto Junior, Rosane Patricia Chaves, Jheymyson de Sousa Cunha, Renan Campos Chisté, Rosinelson da Silva Pena

**Affiliations:** ^1^ Graduate Program of Food Science and Technology (PPGCTA), Institute of Technology (ITEC) Federal University of Pará (UFPA) Belém Pará Brazil; ^2^ Faculty of Pharmacy Federal University of Minas Gerais (UFMG) Belo Horizonte Minas Gerais Brazil; ^3^ Faculty of Food Engineering (FEA), Institute of Technology (ITEC) Federal University of Pará (UFPA) Belém Pará Brazil

**Keywords:** activation energy, diffusivity, *Manihot esculenta*, mathematical modeling, thin‐layer drying

## Abstract

This study investigated the convective drying kinetics of cassava ( *Manihot esculenta* Crantz) leaves from three varieties (*Manipeba*—M1, *Tareza 1*—M2, and *Folha fina*—M8). Drying experiments were conducted in a tray dryer with forced air circulation at temperatures ranging from 40°C to 80°C, and the process was monitored until the sample mass reached equilibrium. The results highlighted that temperatures above 50°C (60°C, 70°C, and 80°C) significantly enhance water diffusion in cassava leaves, leading to reduced drying times. The effective diffusivity (D_eff_) of M1 (1.15–6.32 × 10^−9^), M2 (1.67–7.73 × 10^−9^), and M8 (1.77–7.97 × 10^−9^ m^2^/s) increased with temperature. The higher activation energy (E_a_) of sample M1 (39.33 kJ/mol) suggests greater temperature sensitivity of water diffusivity compared with samples M2 (35.71 kJ/mol) and M8 (36.50 kJ/mol). Nine mathematical models were fitted to the drying experimental data, and the Page and Midilli models best described the drying curves (*R* ^2^ > 0.99, RMSE < 0.05). The findings contribute to understanding the drying behavior of cassava leaves and define conditions for the drying process.

## Graphical abstract

The graphical abstract summarizes the main steps and key findings of the study in a simplified manner.

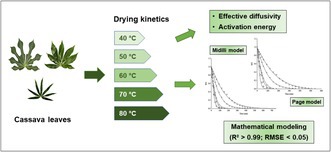

## 1 Introduction

Cassava ( *Manihot esculenta* Crantz) is a globally significant crop due to its adaptability to diverse environments. It is cultivated in all states of Brazil because of its low soil fertility requirements (Tironi et al. 2015). This adaptability has made cassava one of the major food sources in recent years, with Africa leading in production; the largest producers are Nigeria (the world's leading producer), the Democratic Republic of the Congo, and Ghana. Asia is the second‐largest producing region worldwide, while Brazil ranks fourth (FAO (Food and Agriculture Organization of the United Nations) 2017; Santos et al. 2020). Consequently, small farmers widely cultivate cassava, mainly for subsistence, especially in developing countries, as this crop is relatively simple to grow (Pinheiro et al. 2021).

Currently, the aerial parts of cassava are used in animal feed due to their high protein content, and the leaves and stems are also widely utilized in ethanol production. In some regions of Brazil, the leaves are used in human food, especially in typical dishes from the North and Northeast (Fahima et al. 2023). Regarding nutritional value, cassava leaves are recognized as good sources of proteins, fibers, vitamins, minerals, and bioactive compounds (Latif and Müller 2015; Bayata 2019). Thus, when properly processed, cassava leaves can provide significant nutritional benefits to the diet.

The leaves are harvested with a high‐water content, which increases the risks of contamination, deterioration, and enzymatic reactions during storage (Alegbeleye et al. 2022). Therefore, drying can be employed to ensure the quality and stability of the leaves during storage, as this process reduces water content, thereby decreasing biological activities and physical and chemical changes (Vidinamo et al. 2021; Chaiareekitwat et al. 2025).

In addition, the reduction of HCN may be related to water loss during drying, which interrupts the enzymatic activity responsible for hydrolysis and, consequently, decreases the conversion of cyanogenic glycosides into HCN (Ndubuisi and Chidiebere 2018; Modesto Junior et al. 2019; Chaiareekitwat et al. 2025; Forkum et al. 2025). This effect makes cassava leaves safer for human consumption and supports their use as both a food and a medicinal herb (Okareh et al. 2021; Jumare et al. 2024).

Convective drying is a complex mechanism that involves simultaneous heat and mass transfer, as well as the movement of liquids and vapors. During this process, chemical transformations occur in the compounds that influence the properties of the material. The effectiveness of these transformations depends directly on the control of drying temperature and can have either positive or negative effects on the nutritional quality and sensory characteristics of the product, as well as on the drying mechanisms (Chandramohan 2020; Tarafdar et al. 2021).

The behavior of the transfer processes can be described by mathematical models that represent drying, providing valuable information for equipment development and predicting drying conditions (Tarafdar et al. 2021). However, simulating the drying mechanism in a thin layer of a product requires adopting a model that accurately describes water loss during the drying process (Giner and Mascheroni 2002).

Three types of models are commonly used to describe drying kinetics: empirical and semi‐theoretical models, which rely on external variables such as temperature and relative humidity of the drying air; and theoretical models, which consider internal resistance and heat and mass transfer between the product and the drying air (Midilli et al. 2002; Panchariya et al. 2002; Gomes et al. 2022).

Therefore, this study aimed to obtain drying curves in a tray drying oven for leaves of three cassava varieties at different temperatures ranging from 40°C to 80°C, determine the effective diffusion coefficient and activation energy for the drying process, and evaluate the fit of mathematical models to the drying data. Although cassava leaves are traditionally used in regional dishes in the Amazon, few studies have examined their behavior during the drying process, which motivated this research.

## 2 Material and Methods

### 2.1 Plant Material

The cassava ( *Manihot esculenta* Crantz) leaves used in this study were collected after 6 months of planting, in a cassava farm from Salvaterra (Marajó, Pará, Brazil). Leaves from three cassava varieties were collected and identified through exsiccates by the Department of Botanical Identification of Embrapa Amazonia Oriental (Brazilian Agricultural Research Corporation (EMBRAPA)—EMBRAPA Eastern Amazon Research Center) (Belém, Pará, Brazil). For better understanding, the three cassava varieties were identified as *Manipeba* (M1), *Tareza 1* (M2), and *Folha Fina* (M8).

At the Federal University of Pará (UFPA), cassava leaves were selected, with torn, darkened, or yellowed leaves discarded for each variety. The leaves were sanitized by immersion in a sodium hypochlorite solution (100 mg/L active chlorine) for 15 min. Excess water was then drained, and the leaves were ground in an analytical mill (IKA, A11 basic, Germany), homogenized, and used in the drying experiments (Pahariya et al. 2022; Chaves et al. 2023).

### 2.2 Drying of Cassava Leaves

The ground leaves were subjected to convective drying in a tray dryer with forced air circulation (Quimis, Q315M5, Diadema, Brazil) at 40°C, 50°C, 60°C, 70°C, and 80°C (Chaiareekitwat et al. 2025), with an average air velocity of 1.0 m/s. For the drying experiments, approximately 10 g samples were evenly distributed in aluminum containers with a known mass to form a thin layer. The samples were weighed in a semi‐analytical balance (±0.001 g) (Bel‐Engineering—M214i, São Paulo, Brazil) every 10 min for the first 30 min; every 15 min for the next 45 min; and every 30 min thereafter until the mass reached equilibrium (mass variation less than 1%) (Chaves et al. 2023). At the end of the process, the dry mass of the sample was determined at 105°C (AOAC International 1997). Experiments were conducted in duplicate to ensure reproducibility. The sample moisture content was calculated using Equation (1), and the drying curves were constructed based on the correlation between the moisture ratio (MR) (Equation 2) and the process time (Macedo et al. 2025).

(1) $$m=\frac{M-\mathrm{DM}}{\mathrm{DM}}$$

(2) $$\mathrm{MR}=\frac{m-m_{e}}{m_{i}-m_{e}}$$

where M = mass of the sample at time t (g); DM = dry matter (g); MR = moisture ratio (dimensionless); m, m_i_, and m_e_ = moisture content at time t, initial moisture content, and equilibrium moisture content (g/g dry basis—db), respectively.

The drying rate (DR) was calculated using Equation (3). The value of dm/dt was obtained from the first derivative of the linear equation fitted to the experimental data for the initial linear region of the drying curve (constant drying‐rate period). For the nonlinear region (falling drying‐rate period), dm/dt was determined from the first derivative of the Page equation (Table 1), also fitted to the experimental drying data (Macedo et al. 2025).

(3) $$\mathrm{DR}=\frac{\mathrm{DM}}{A}\left(-\frac{\mathrm{dm}}{\mathrm{dt}}\right)$$

where DR = drying rate (g/m^2^h); DM = dry matter (g); A = drying area (m^2^); m = moisture content at time t (g/g db); t = drying time (h).

**TABLE 1:** Mathematical models used in fitting the drying data of cassava leaves.

Model	Equation^a^	Number of parameters
Newton	$\mathrm{MR}=e^{-\mathrm{kt}}$	1
Page	$\mathrm{MR}=e^{-kt^{n}}$	2
Modified Page	$\mathrm{MR}=e^{-{\left(\mathrm{kt}\right)}^{n}}$	2
Henderson and Pabis	$\mathrm{MR}=ae^{-\mathrm{kt}}$	2
Two‐term exponential	$\mathrm{MR}=ae^{-\mathrm{kt}}+\left(1-a\right)e^{-\mathrm{kat}}$	2
Logarithmic	$\mathrm{MR}=ae^{-\mathrm{kt}}+c$	3
Diffusion approximation	$\mathrm{MR}=ae^{-\mathrm{kt}}+\left(1-a\right)e^{-\mathrm{kbt}}$	3
Midilli	$\mathrm{MR}=\mathrm{bt}+{\mathrm{ae}}^{-kt^{n}}$	4
Two‐term	$\mathrm{MR}=ae^{-k_{0}t}+be^{-k_{1}t}$	4

^a^ Inyang et al. (2018).

### 2.3 Calculation of Effective Diffusivity and Activation Energy

For calculating the effective water diffusivity in the drying process, Fick's second law of diffusion under nonsteady‐state conditions was utilized. This equation, expressed in Cartesian coordinates and dimensionless form, can be written as shown in Equation (4) (Crank 1975).

(4) $$\frac{\mathrm{\partial MR}}{\mathrm{\partial t}}=\frac{\partial }{\mathrm{\partial y}}\left(D_{\mathrm{eff}}\frac{\mathrm{\partial MR}}{\mathrm{\partial y}}\right)$$

where D_eff_ = effective diffusivity; t = time; y = rectangular coordinate; MR = moisture ratio.

According to Crank (1975), the analytical solution of Equation (4), assuming uniform initial moisture content, constant effective diffusivity throughout the sample, and samples with slab geometry, is given by Equation (5). For sufficiently long drying times, the first term in the series expansion provides a good estimate of the solution (Equation 6). The D_eff_ values were calculated from the slopes of curves plotted as ln(MR) values versus drying time (t).

(5) $$\mathrm{MR}=\frac{8}{\pi^{2}}\sum_{i=0}^{\infty }\frac{1}{{\left(2i+1\right)}^{2}}\exp\left[\frac{-{\left(2i+1\right)}^{2}\pi^{2}D_{\mathrm{eff}}}{4L^{2}}t\right]$$

(6) $$\mathrm{MR}=\frac{m-m_{e}}{m_{i}-m_{e}}=\frac{8}{\pi^{2}}\exp\left(\frac{-\pi^{2}D_{\mathrm{eff}}}{4L^{2}}t\right)$$

where MR = moisture ratio (dimensionless); m, m_i_ and m_e_ = moisture content at time t, initial moisture content, and equilibrium moisture content (g/g db), respectively; D_eff_ = effective diffusivity (m^2^/s); L = half‐thickness of the slab (5 mm); t = drying time (s).

The dependence of D_eff_ on temperature can be expressed by an equation analogous to the Arrhenius equation (Equation 7) (Doymaz 2013). This equation was used to determine the activation energy (E_a_) of the drying process from the slope of the Ln(D_eff_) versus the 1/T curve.

(7) $$\mathrm{Ln}\left(D_{\mathrm{eff}}\right)=\ln\left(D_{0}\right)-\frac{E_{a}}{\mathrm{RT}}$$

where D_0_ = pre‐exponential factor of the Arrhenius‐type equation (m^2^/s); E_a_ = activation energy (J/mol); *R* = universal gas constant (8.31 J/K·mol); and T = absolute temperature of the drying air (K).

### 2.4 Mathematical Modeling of Drying

To evaluate drying models, nine semiempirical equations commonly applied in thin‐layer drying were tested (Table 1).

### 2.5 Statistical Analysis

D_eff_ and E_a_ values were calculated using linear regression, while the fitting of mathematical models to the drying data was performed using nonlinear regression. The quality of the fits was evaluated using the coefficient of determination (*R* ^2^), reduced chi‐square (χ^2^) (Equation 8), and root mean square error (RMSE) (Equation 9) (Macedo et al. 2025). Statistical analysis was conducted using Statistic software, version 7.0.

(8) $$\chi^{2}=\frac{\sum_{i=1}^{N}{\left({\mathrm{MR}}_{\exp,i}-{\mathrm{MR}}_{\mathrm{pre},i}\right)}^{2}}{N-n}$$

(9) RMSE=1N∑i=1NMRexp,i−MRpre,i212

where MR_exp,*i*_ and MR_pre,*i*_ = moisture ratio obtained experimentally and predicted by the model, respectively; *N* = number of experimental measurements; *n* = number of parameters of the model.

## 3 Results and Discussion

### 3.1 Drying Kinetics of Cassava Leaves

**FIGURE 1:**
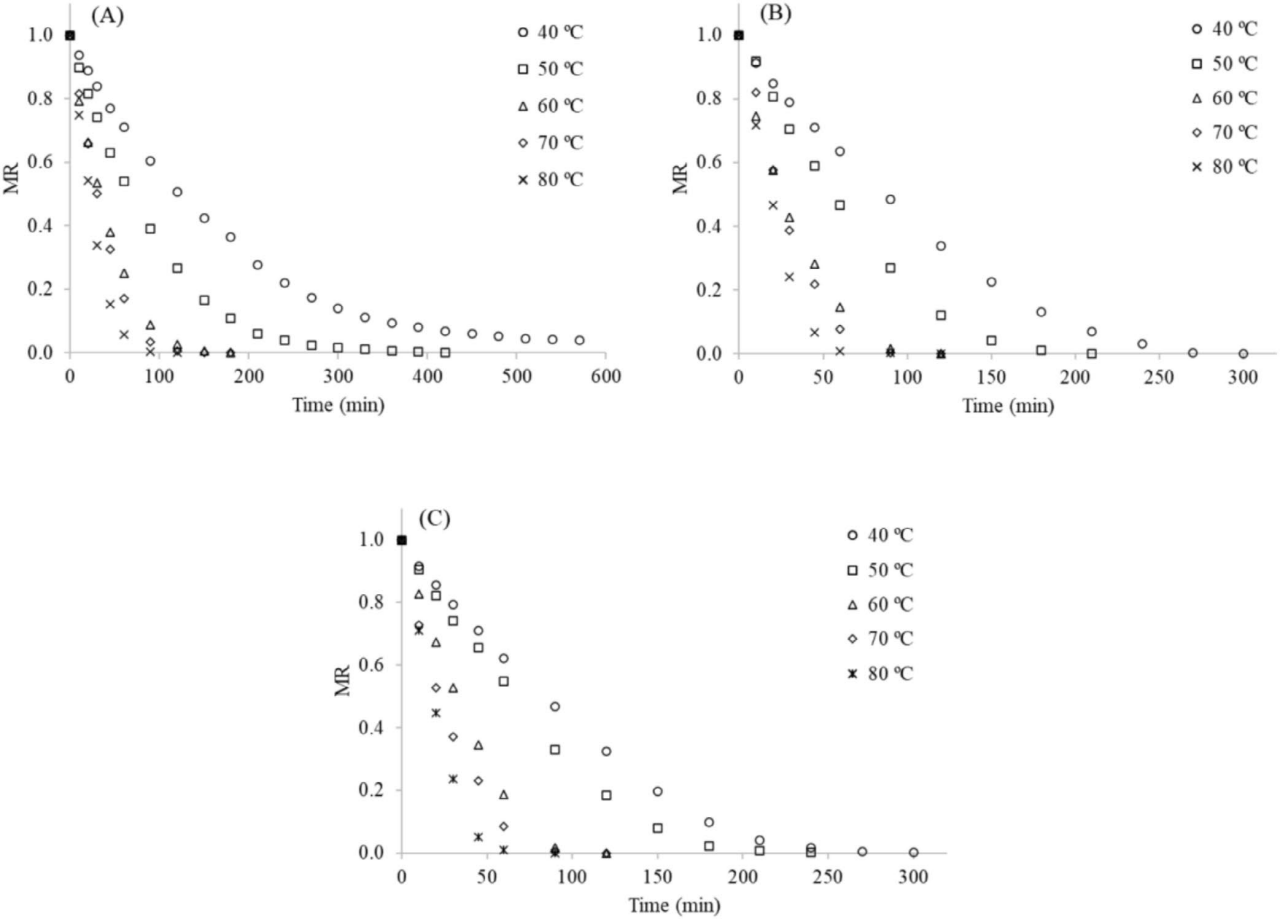
Drying curves of leaves from different cassava varieties: (A) M1, (B) M2, and (C) M8. MR values represent the mean of duplicate measurements, with variability below 5%.

Figure 1 shows the drying curves at different temperatures (40°C–80°C) for the leaves of three cassava varieties. Overall, the drying curves exhibited similar behavior; however, the leaves of variety M1 required longer drying times at all temperatures, ranging from 150 to 570 min at 80°C to 40°C (Figure 1A). In contrast, drying times for variety M2 ranged from 150 to 330 min at 80°C to 40°C (Figure 1B), while those for variety M8 ranged from 120 to 330 min at 80°C to 40°C (Figure 1C).

The slower drying of the M1 variety increases processing times and likely energy consumption, and may also contribute to product degradation. These findings are relevant for industrial applications and cassava variety selection, as faster drying rates can enhance productivity, reduce energy use, and help preserve physical, physicochemical, and chemical quality.

Taking drying at 40°C as a reference, increasing the drying temperature generally reduced the drying time, reaching reductions of 21% for M1, 27% for M2, and 18% for M8 at 50°C; 63% for M1 and 55% for M2 and M8 at 60°C and 70°C; and 74% for M1, 55% for M2, and 64% for M8 at 80°C (Figure 1). These findings are highly relevant for practical applications.

**FIGURE 2:**
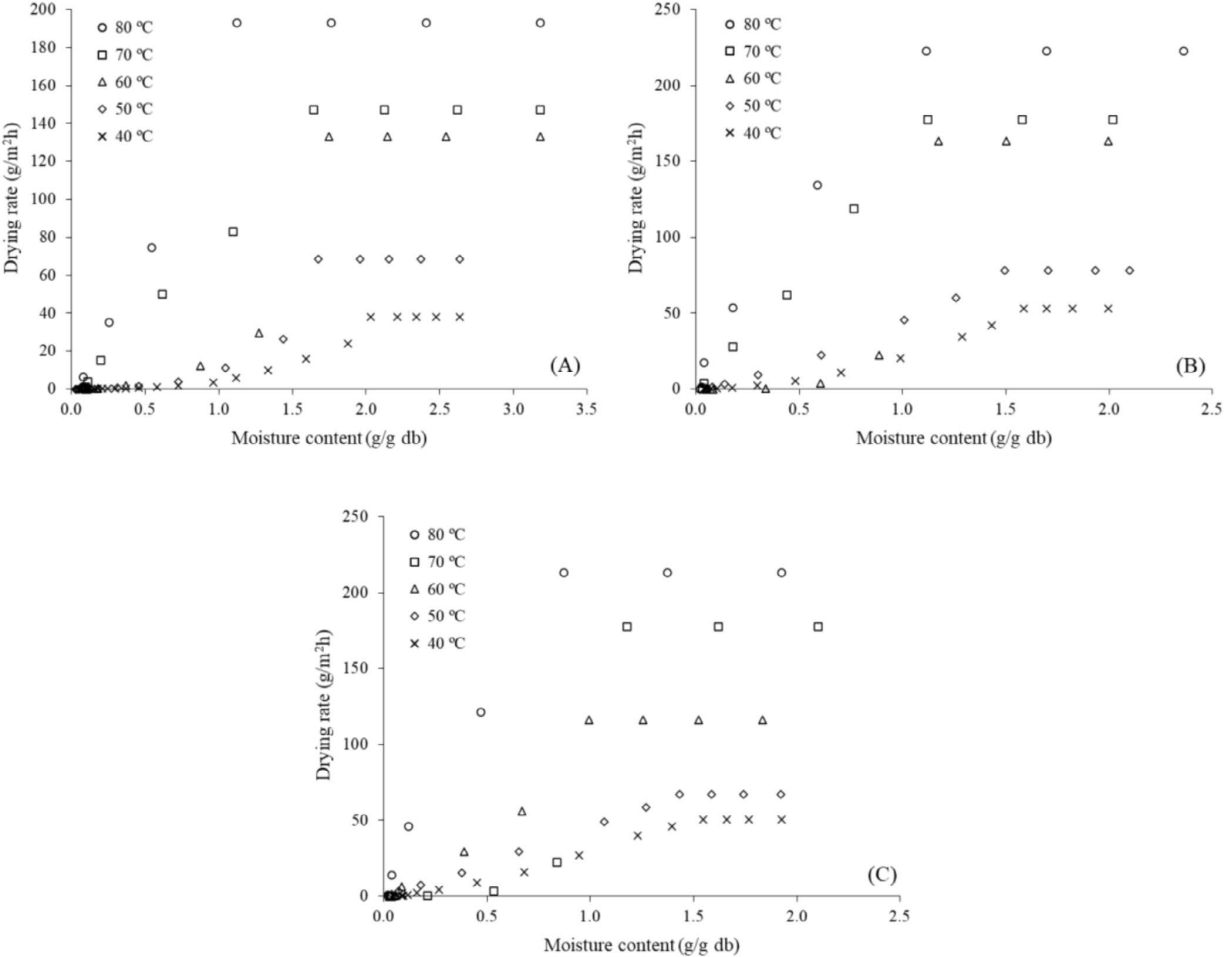
Drying rate curves as a function of moisture content for (A) M1, (B) M2, and (C) M8.

Figure 2 presents the drying‐rate curves as a function of moisture content, in which the different drying stages can be identified. For all varieties (M1, M2, and M8) and drying temperatures, an initial constant drying‐rate period followed by a falling drying‐rate period was observed. In general, the critical moisture content—at which the constant drying‐rate period ends and the falling drying‐rate period begins—decreased with increasing drying temperature.

Overall, the drying rate (DR) values for variety M1 (Figure 2A) were lower than those for varieties M2 (Figure 2B) and M8 (Figure 2C) at the different temperatures, confirming the longer drying times required for variety M1 (Figure 1A). For all varieties (M1, M2, and M8), higher DR values were observed at drying temperatures above 50°C (60°C–80°C).

Chaiareekitwat et al. (2025) investigated the effect of different convective drying temperatures (40°C–80°C) on cassava leaves of the Rayong 5 cultivar. They also found that higher temperatures resulted in shorter drying times and recommended drying at 80°C for approximately 2.5 h. The authors concluded that high drying air temperatures are effective for producing dehydrated cassava leaves with high nutritional quality.

**FIGURE 3:**
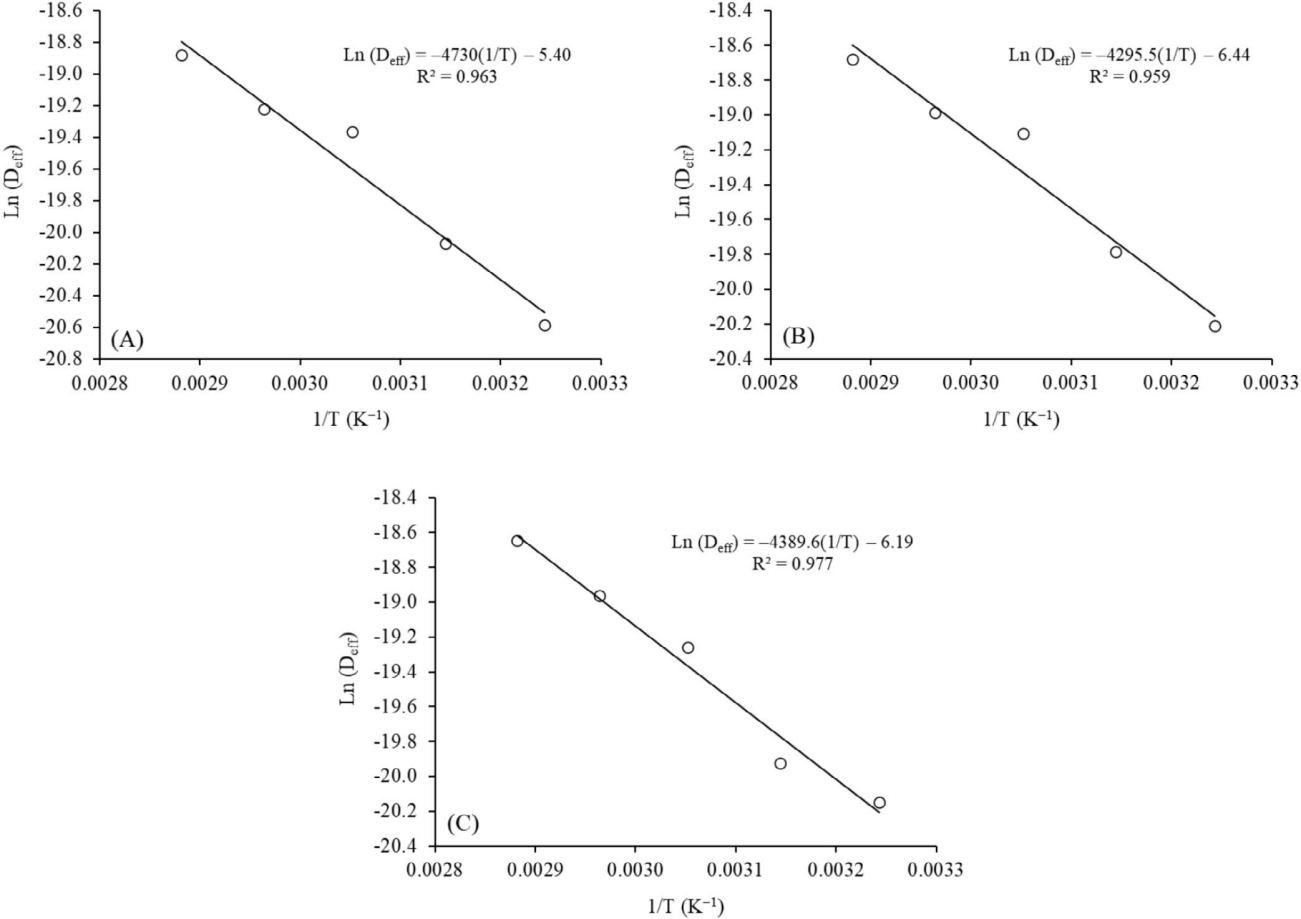
Natural logarithm of the effective diffusivity (D_eff_) as a function of the inverse of the drying temperature for (A) M1, (B) M2, and (C) M8.

Table 2 presents the effective diffusivity (D_eff_) values for the drying of leaves from different cassava varieties at various temperatures, while Figure 3 shows the Ln(D_eff_) versus 1/T plots for each variety, including the fitted equations, the line slopes used to calculate the activation energy (E_a_), and the corresponding *R* ^2^ values.

**TABLE 2:** Effective diffusivity (D_eff_) values for the drying processes of cassava leaves M1, M2, and M8, at different temperatures.

Drying temperature (°C)	Cassava variety/Effective diffusivity		
	M1	M2	M8
	D_eff_ (m^2^/s)		
40	1.15 × 10^−9^	1.67 × 10^−9^	1.77 × 10^−9^
50	1.93 × 10^−9^	2.55 × 10^−9^	2.23 × 10^−9^
60	3.88 × 10^−9^	5.05 × 10^−9^	4.32 × 10^−9^
70	4.48 × 10^−9^	5.67 × 10^−9^	5.83 × 10^−9^
80	6.32 × 10^−9^	7.73 × 10^−9^	7.97 × 10^−9^

*Note:* Varieties: M1—Manipeba, M2—Tareza, and M8—Folha Fina.

The D_eff_ values (Table 2) confirm that, regardless of the variety, increasing the temperature favored water loss, with this effect being more pronounced at temperatures above 50°C (60°C–80°C) (Figure 1). Additionally, the lower D_eff_ values for the leaves of variety M1 confirm that the drying rates were lower for this variety (Figure 2), possibly due to the physicochemical properties of the leaves. According to Modesto Junior et al. (2019), the M1 variety has the highest lipid content in its composition, which can interfere with the water loss processes.

According to Goneli et al. (2014), increasing the drying temperature reduces the viscosity of water, which is a measure of fluid resistance. Decreased viscosity promotes the diffusion of water within the capillaries of the leaves. The increase in D_eff_ with rising drying temperatures can also be attributed to the heightened vibration intensity of water molecules, thereby enhancing water diffusion. D_eff_ values of the same order of magnitude (1.08–6.35 × 10^−9^ m^2^/s) were observed for the convective drying of *Inga edulis* leaves in the temperature range of 40°C–70°C (Silva et al. 2011).

Chaves et al. (2023) observed lower values of D_eff_ for the tray drying of purple basil ( *Ocimum basilicum* L.) leaves being 4.93 × 10^−10^ m^2^/s at 40°C, 9.94 × 10^−10^ m^2^/s at 50°C, 13.87 × 10^−10^ m^2^/s at 60°C, and 18.96 × 10^−10^ m^2^/s at 70°C. Mbegbu et al. (2021) observed D_eff_ values for the drying of sweet basil ( *Ocimum gratissimum* ) leaves (4.76 × 10^−13^—1.47 × 10^−12^ m^2^/s) and lemon basil ( *Ocimum basilicum* ) leaves (4.83 × 10^−13^—2.06 × 10^−12^ m^2^/s), for both process carried out in a vacuum oven at temperatures between 30°C and 70°C. These values are approximately 1000 times lower than those observed for cassava leaf drying, indicating significantly lower water diffusion resistance in cassava leaves.

Therefore, the D_eff_ values, drying curves (Figure 1), and drying rate curves (Figure 2) obtained in this study provide valuable guidance for the design and operation of industrial dryers, enabling adjustments to processing conditions that optimize water removal without compromising final product quality. Producers and processing industries are advised to prioritize cassava varieties with higher drying efficiency and to adopt operational conditions that balance processing time, energy consumption, and the preservation of the leaves' overall characteristics (Oliveira et al. 2016; ElGamal et al. 2023).

The activation energy (E_a_) values were 39.33 for the drying of variety M1, 35.71 for variety M2, and 36.50 kJ/mol for variety M8. According to Yao et al. (2008), E_a_ values between 20 and 50 kJ/mol are generally reported for fiber‐rich plant materials, in which net diffusion occurs predominantly through the compact cell matrix.

The highest E_a_ value observed to M1 (39.33 kJ/mol) indicates that moisture diffusion in leaves of this cassava variety requires a greater energy input. In contrast, the lowest E_a_ values for M2 (35.71 kJ/mol) and M8 (36.50 kJ/mol) indicates that the leaves of these varieties present a lower energetic barrier for the diffusional process. Therefore, the higher E_a_ observed for variety M1 suggests greater internal resistance to moisture diffusion, which may be associated with more rigid microstructures, greater leaf thickness, and higher compaction of parenchymal tissue (Yao et al. 2008).

### 3.2 Mathematical Modeling of Drying Data

The statistical criteria using coefficient of determination (*R* ^2^), reduced chi‐square (χ^2^), and root mean square error (RMSE) were evaluated to assess the best fit of the drying models as shown in Table 3. Values of *R* ^2^ greater than 0.98 were obtained for the nine models fitted to all drying runs, indicating a good description of the drying processes. Additionally, the low values of χ^2^ (< 0.003) and RMSE (< 0.05) confirm the models' accuracy in representing the drying behavior. They further confirm that all models were capable of predicting the drying kinetics of cassava leaves with good accuracy within the experimental domain.

**TABLE 3:** Values of parameters for fitting mathematical models to cassava leaf drying data.

Model	Temperature (°C)	M1			M2			M8		
		*R* ^2^	χ^2^ × 10^4^	RMSE	*R* ^2^	χ^2^ × 10^4^	RMSE	*R* ^2^	χ^2^ × 10^4^	RMSE
Newton	40	0.993	1.95	0.013	0.979	0.11	0.032	0.977	0.32	0.054
	50	0.996	1.72	0.012	0.978	0.19	0.041	0.975	0.35	0.056
	60	0.994	5.49	0.023	0.994	7.66	0.025	0.997	0.30	0.051
	70	0.987	16.30	0.038	0.980	0.28	0.049	0.994	7.37	0.025
	80	0.987	18.10	0.039	0.982	25.80	0.047	0.980	0.29	0.049
Page	40	0.997	1.11	0.010	0.994	5.56	0.021	0.994	8.03	0.026
	50	0.999	0.85	0.008	0.997	2.81	0.015	0.995	7.22	0.024
	60	0.997	3.35	0.016	0.996	5.44	0.020	0.996	5.92	0.021
	70	0.998	2.10	0.012	0.999	1.44	0.010	0.997	4.20	0.017
	80	0.999	1.45	0.010	0.998	2.12	0.012	0.998	2.32	0.012
Modified Page	40	0.997	1.11	0.010	0.994	5.56	0.021	0.994	8.03	0.026
	50	0.999	0.85	0.008	0.997	2.81	0.015	0.995	7.22	0.024
	60	0.997	3.35	0.016	0.996	5.44	0.020	0.996	5.92	0.021
	70	0.998	2.10	0.012	0.999	1.44	0.010	0.997	4.20	0.017
	80	0.999	1.45	0.010	0.998	2.12	0.012	0.999	2.32	0.012
Henderson and Pabis	40	0.994	1.91	0.013	0.982	0.11	0.031	0.981	0.28	0.049
	50	0.996	1.74	0.012	0.984	0.15	0.036	0.980	0.31	0.050
	60	0.994	6.34	0.022	0.994	8.61	0.025	0.981	0.29	0.047
	70	0.990	15.10	0.035	0.984	0.03	0.044	0.994	8.05	0.024
	80	0.988	18.2	0.037	0.984	0.26	0.044	0.982	0.31	0.047
Two‐term Exponential	40	0.997	0.67	0.008	0.993	4.70	0.020	0.993	9.86	0.029
	50	0.999	0.55	0.007	0.996	2.66	0.014	0.994	9.45	0.028
	60	0.997	2.85	0.015	0.996	5.01	0.019	0.994	8.09	0.024
	70	0.998	2.78	0.015	0.998	2.05	0.012	0.997	4.06	0.017
	80	0.998	2.26	0.013	0.997	4.32	0.018	0.997	4.81	0.018
Logarithmic	40	0.998	0.70	0.007	0.996	5.13	0.020	0.994	8.76	0.026
	50	0.998	0.67	0.007	0.995	3.99	0.017	0.993	0.10	0.028
	60	0.997	3.25	0.015	0.998	6.02	0.019	0.994	9.70	0.024
	70	0.993	3.18	0.014	0.990	2.46	0.012	0.997	4.87	0.017
	80	0.992	2.71	0.013	0.988	5.18	0.018	0.989	6.01	0.018
Diffusion Approximation	40	0.998	0.67	0.007	0.994	4.68	0.019	0.994	9.11	0.026
	50	0.999	0.55	0.006	0.997	3.31	0.015	0.995	8.74	0.025
	60	0.998	2.95	0.014	0.995	5.06	0.018	0.995	8.06	0.022
	70	0.998	2.99	0.013	0.998	2.05	0.011	0.997	4.52	0.016
	80	0.998	2.00	0.011	0.998	3.96	0.015	0.997	4.75	0.016
Midili	40	0.998	0.48	0.006	0.998	2.39	0.013	0.997	4.38	0.017
	50	0.999	0.63	0.007	0.998	1.72	0.010	0.997	4.91	0.018
	60	0.998	3.01	0.013	0.982	3.99	0.014	0.997	4.89	0.015
	70	0.998	1.74	0.010	0.999	1.95	0.009	0.998	4.51	0.015
	80	0.999	1.93	0.009	0.998	2.97	0.012	0.998	3.35	0.011
Two‐term	40	0.994	0.74	0.007	0.982	5.64	0.020	0.995	8.00	0.023
	50	0.996	0.67	0.007	0.984	4.56	0.017	0.994	0.11	0.028
	60	0.994	3.80	0.015	0.994	7.52	0.019	0.995	0.12	0.024
	70	0.990	3.74	0.014	0.984	3.07	0.012	0.998	6.09	0.017
	80	0.988	3.39	0.013	0.984	6.48	0.018	0.982	8.01	0.018

*Note:* Varieties: M1—*Manipeba*, M2—*Tareza*, and M8—*Folha Fina*.

**FIGURE 4:**
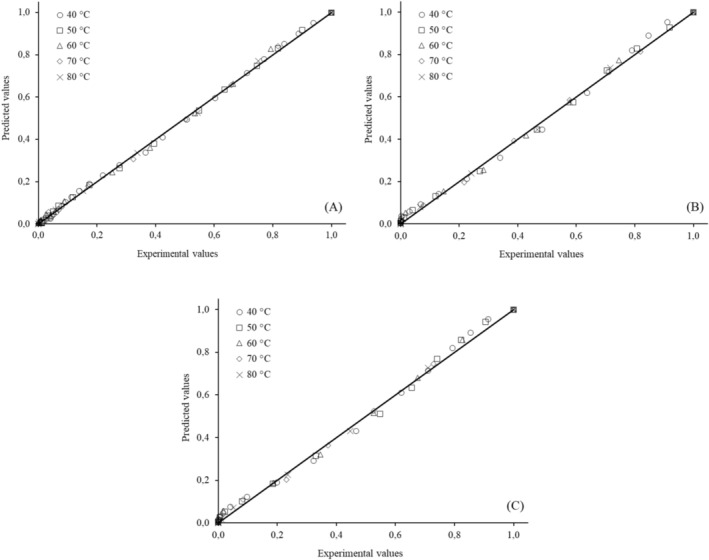
Experimental moisture ratio plotted against the values predicted by the Page model for (A) M1, (B) M2, and (C) M8.

**FIGURE 5:**
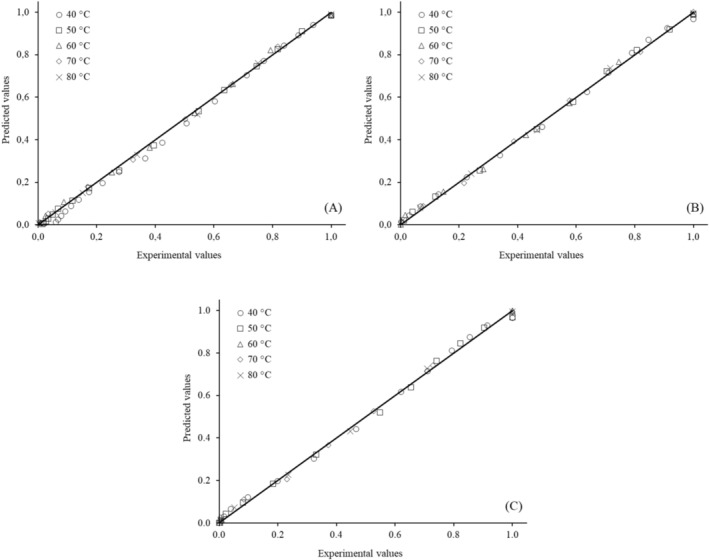
Experimental moisture ratio plotted against the values predicted by the Midilli model for (A) M1, (B) M2, and (C) M8.

However, the Page (*R* ^2^ > 0.99; χ^2^ < 0.0008 and RMSE < 0.05) and Midilli (*R* ^2^ > 0.99; χ^2^ < 0.0005 and RMSE < 0.02) models showed the best performance across all studied conditions, as evidenced by the strong correlations between the experimental and predicted moisture rate (MR) values in Figures 4 and 5. The Page model is biparametric, whereas the Midilli model has four parameters. Therefore, due to its simpler mathematical solution, the Page model is recommended for predicting the drying behavior of cassava leaves under the studied conditions.

Chaiareekitwat et al. (2025) identified the Page model as the most suitable for describing the drying behavior of cassava leaves of the Rayong 5 variety. Barbosa et al. (2007), who dried Brazilian lemon‐scented verbena (*Lippi alba* [Mill] N.E. Brown) leaves in a fixed‐bed dryer at temperatures ranging from 40°C to 80°C, reported that the Page and Midilli models best represented the drying curves of the leaves.

In studies on the drying of leaves of other plant species, such as wolf fruit ( *Solanum lycocarpum* ) in a fixed bed (Prates et al. 2012), *Gundelia tourneforti* L. in thin‐layer drying (Evin 2012), and purple basil ( *Ocimum basilicum* L.) in a tray dryer (Chaves et al. 2023), authors observed that the Modified Page, Diffusion Approximation, and Verna models were efficient in predicting the drying kinetics.

For basil leaves ( *Ocimum basilicum* L.) using infrared drying, Reis et al. (2012) observed that the Midilli model provided the best fit to the drying data. According to Goneli et al. (2014), the Midilli model fits well with the drying data of leaves because, for these types of products, the drying process involves rapid water loss in the initial stage, similar to what was observed for cassava leaves, especially at temperatures above 50°C (60°C–80°C) (Figure 2).

## 4 Conclusion

The drying rate of cassava leaves was strongly influenced by both variety and temperature, particularly at temperatures above 50°C (60°C–80°C). The *Manipeba*—M1 variety exhibited lower drying rate, whereas the *Tareza 1*—M2 and *Folha fina*—M8 varieties dried more effectively, which can improve productivity and reduce energy consumption. The Page and Midilli models provided excellent fits to the drying data and can be reliably used to predict cassava leaf drying curves within the experimental domain. Although several studies have investigated different cassava leaf varieties, further research is needed to expand current knowledge, particularly by exploring alternative drying methods and evaluating their effects on nutritional quality, bioactive compound retention, and the feasibility of industrial‐scale processing.

## Author Contributions

**Elivaldo Nunes Modesto Junior:** conceptualization, data curation, formal analysis, investigation, methodology, software, writing – original draft. **Rosane Patricia Chaves:** formal analysis, methodology, software, writing – original draft. **Jheymyson de Sousa Cunha:** formal analysis, methodology, software, writing – original draft. **Renan Campos Chisté:** conceptualization, data curation, formal analysis, supervision, validation, visualization, writing – review and editing. **Rosinelson da Silva Pena:** conceptualization, data curation, formal analysis, project administration, resources, supervision, validation, visualization, writing – review and editing.

## Funding

This work was supported by the *Coordenação de Aperfeiçoamento de Pessoal de Nível Superior* (CAPES, Brazil) through a scholarship awarded to Elivaldo N. Modesto Junior (Grant number 88882.445440/2019‐01).

## Conflicts of Interest

The authors declare no conflicts of interest.

## Data Availability

The data that support the findings of this study are available from the corresponding author upon reasonable request.
